# Changes in striatal dopamine transporters in bipolar disorder and valproate treatment

**DOI:** 10.1192/j.eurpsy.2021.1

**Published:** 2021-01-08

**Authors:** Yuan-Shuo Hsueh, Chih-Ying Lin, Nan-Tsing Chiu, Yen Kuang Yang, Po See Chen, Hui Hua Chang

**Affiliations:** 1 Department of Medical Science Industries, College of Health Sciences, Chang Jung Christian University, Tainan, Taiwan; 2 Department of Biotechnology and Bioindustry Sciences, National Cheng Kung University, Tainan, Taiwan; 3 Institute of Clinical Pharmacy and Pharmaceutical Sciences, College of Medicine, National Cheng Kung University, Tainan, Taiwan; 4 Department of Nuclear Medicine, National Cheng Kung University Hospital, College of Medicine, National Cheng Kung University, Tainan, Taiwan; 5 Department of Psychiatry, National Cheng Kung University Hospital, College of Medicine, National Cheng Kung University, Tainan, Taiwan; 6 Department of Psychiatry, National Cheng Kung University Hospital Dou-Liou Branch, Dou-Liou, Yunlin, Taiwan; 7 Institute of Behavioral Medicine, College of Medicine, National Cheng Kung University, Tainan, Taiwan; 8 School of Pharmacy, College of Medicine, National Cheng Kung University, Tainan, Taiwan; 9 Department of Pharmacy, National Cheng Kung University Hospital, College of Medicine, National Cheng Kung University, Tainan, Taiwan; 10 Department of Pharmacy, National Cheng Kung University Hospital, Dou-Liou Branch, Yunlin, Taiwan

**Keywords:** Bipolar disorder, dopamine transporter, striatum, valproate

## Abstract

**Background:**

Previous studies suggested that a disturbance of the dopamine system underlies the pathophysiology of bipolar disorder (BD). In addition, the therapeutic action of medications for treating BD, such as valproate (VPA), might modulate dopamine system activity, but it remains unclear. Here, we aimed to investigate the role of the striatal dopamine transporter (DAT) in BD patients and in social defeat (SD) mice treated with VPA.

**Methods:**

We enrolled community-dwelling controls (*N* = 18) and BD patients (*N* = 23) who were treated with VPA in a euthymic stage. The striatal DAT availabilities were approached by TRODAT-1 single photon emission computed tomography. We also established a chronic SD mouse model and treated mice with 350 mg/kg VPA for 3 weeks. Behavioral tests were administered, and striatal DAT expression levels were determined.

**Results:**

In humans, the level of striatal DAT availability was significantly higher in euthymic BD patients (1.52 ± 0.17 and 1.37 ± 0.23, *p* = 0.015). Moreover, the level of striatal DAT availability was also negatively correlated with the VPA concentration in BD patients (*r* = −0.653, *p* = 0.003). In SD mice, the expression of striatal DAT significantly increased (*p* < 0.001), and the SD effect on DAT expression was rescued by VPA treatment.

**Conclusions:**

The striatal DAT might play a role in the pathophysiology of BD and in the therapeutic mechanism of VPA. The homeostasis of DAT might represent a new therapeutic strategy for BD patients.

## Introduction

The dopaminergic pathway has long been known to be involved in the regulation of emotion, cognitive function, reward, and motor function [1,2]. In the dopamine system, the dopamine transporter (DAT) plays a key role in the dopaminergic system by regulating the reuptake of extracellular dopamine into presynaptic neurons, and the dysfunction of the DAT can lead to abnormal mood and behavior [3,4]. Medications affecting DAT function, such as cocaine and amphetamines, result in hyperactivity and behavioral changes by enhancing dopaminergic signaling. The disturbance of the dopamine system has been reported to be associated with bipolar disorder (BD) [2,5]. Our previous study demonstrated elevated striatal DAT availability and unchanged dopamine D2 receptor binding potential in untreated euthymic BD patients compared with controls [6]. In addition, human genetic studies have shown that polymorphisms of the *DAT* gene play roles in the etiology of BD [7,8].

Mood stabilizers, such as valproate (VPA) and lithium, are usually the first choice to treat BD. A study using the dopaminergic N27 cell line indicated that VPA increases DAT expression through histone acetylation and enhances promoter binding of Nurr1 [9]. In human studies, decreased presynaptic dopamine function was found in manic patients treated with VPA, while there was no change in D2/D3 receptor availability [10,11]. However, whether DAT availability is influenced in euthymic BD patients treated with VPA remains unclear. In this study, therefore, we aimed to investigate not only the association between BD itself and DAT availability but also the correlation between VPA levels and striatal DAT availability in euthymic BD patients. In addition, we used a chronic social defeat (SD) mouse model to test whether striatal DAT expression changes after VPA treatment.

## Materials and Methods

### Human study

#### Participants

The Institutional Review Board for the Protection of Human Subjects at National Cheng approved the research protocol. This study was conducted in accordance with the Declaration of Kung University Hospital Helsinki. All participants were recruited from outpatient settings at the National Cheng Kung University Hospital (NCKUH; NCT04486092) and provided written informed consent regarding their willingness to participate in the research. The inclusion criteria for the 23 BD patients were as follows: (a) diagnosed by experienced attending psychiatrists in accordance with the Diagnostic and Statistical Manual of Mental Disorders, Fifth Edition (DSM-5) criteria for bipolar II disorder, (b) 18–65 years of age, (c) receiving VPA (Depakine®, Carbon Blanc, France) treatment, and (d) in a euthymic stage as measured by the 11-item Young Mania Rating Scale (YMRS) ≤ 7 and the 17-item Hamilton Rating Scale for Depression (HDRS) ≤ 7. The exclusion criteria were as follows: (a) substance or alcohol abuse; (b) organic mental disorder, intellectual disability, neurological illness, neurocognitive disorder; and (c) pregnancy or breastfeeding. To assess whether BD influences the correlations, we recruited healthy controls from the community after excluding individuals with mental illnesses, as measured by a senior psychiatrist using the Chinese version of the Mini International Neuropsychiatry Interview.

In this study, BD patients received VPA in the therapeutic range of 40–120 μg/mL. The BD patients underwent treatment with VPA for at least 3 months. Concomitant fluoxetine (Prozac®, Bourgoin-Jallieu, France) (20 mg/day) was permitted to treat depressive symptoms, and lorazepam (Ativan®, Munster, Germany) (<8 mg) was used for night-time sedation and to treat agitation and insomnia during the study, the dosage of which was adjusted according to the clinical manifestation and the patient’s tolerance. Fasting blood samples were collected between 08:00 and 10:00 am. Serum trough concentration of VPA was assessed by the homogeneous enzyme immunoassay method at the laboratory of the Pathology Research Center of NCKUH.

#### Brain imaging

Striatal DAT availability was measured by single photon emission computed tomography. For brain imaging, each participant was intravenously administered 740 MBq (20 mCi)-TRODAT-1 (a radiolabeled form of a tropan derivative for the selective labeling of DAT, Lung-Tan, Taiwan) [12]. The ratio of the radioactivity [(St − Oc)/Oc ratio] was derived by dividing the difference between the average counts per pixel in the striatum and the average counts per pixel in the occipital cortex by the average counts per pixel in the occipital cortex.

### Animal study

#### Mouse

This study complied with the National Institutes of Health Guide for the Care and Use of Laboratory Animals (NIH Publication No. 80-23) revised in 1996. All experiments were performed according to the National Institutes of Health Guideline for Animal Research (Guide for the Care and Use of Laboratory Animals) and were approved by the National Cheng Kung University (NCKU) Institutional Animal Care and Use Committee. Seven-week-old male C57BL/B6N mice were purchased from BioLASCO (Taipei, Taiwan), and CD1 mice were bred at less than 4 months of age from the NCKU Animal Center. C57BL/B6N mice were housed at a density of 4–5 mice per cage for 1 week, and CD1 mice were singly housed throughout. All mice were housed in a temperature- and humidity-controlled room (25°C) on a 13 h/11 h light/dark cycle with lights on at 07:00 am. The mice had free access to a standard chow diet (Cat #5010, LabDiet, St. Louis, MO) and water. All experiments were performed during the light cycle.

#### Chronic SD mouse model and VPA administration

Previous studies have reported that SD mouse is a useful model for depression, and further evidences showed that chronic SD stress mouse model has BD depression relevant behavior, as depressive-like behavior, hypo-or hyper-activity, reduced exploration, increased or decreased aggression, elevated anxiety, and social avoidance [13–16]. Stressful events in the early period of life produce long-lasting effects on brain development in rodents, comparable to the effects observed in BD patients [16]. A modified protocol for repeated SD stress in mice was followed [17]. CD-1 male mice, selected on the basis of their attack latency (shorter than 600 s) and bouts of attack per 10 min (more than three bouts of attack per 10 min on three consecutive screening tests), were used as aggressors. C57BL/6 N mice were exposed to a different unfamiliar CD-1 aggressor each day for 10 min of full interaction in CD-1 mouse home cages for 10 days. During this exposure, all C57BL/6 N mice showed signs of subordination (i.e., sideways, upright postures, fleeing, or freezing). After 10 min, C57BL/6 N mice were separated from the aggressor by a Plexiglas divider perforated with small holes to allow sensory contact. The mice were housed in this way for the next 24 h, with food and water provided ad libitum. Mice under SD (*N* = 16) or not (*N* = 16) were administered an intraperitoneal injection of normal saline (control; *N* = 8) or 350 mg/kg VPA (Sigma-Aldrich, P4543, St Louis, MO, U.S.A.; *N* = 8) adjusted according to body weight for 3 weeks (8 weeks to 11 weeks old).

#### Social interaction score

Mice were separated and housed individually the night before the experiment to enhance later social interactions. Mice were matched in terms of their weight. After a 60-min habituation period in the room, pairs of either treated or control mice were put into the apparatus (50 cm × 40 cm × 40 cm) over a period of 20 min. The percentage of time spent following, mounting, grooming each other, and sniffing of any body part were considered indicators of social engagement.

#### Forced swimming test

The measurement of the forced swimming test (FST) was assayed as previously described [18]. Mice were individually placed in a clear acrylic plastic cylinder (25 cm diameter × 41 cm height) containing water at a depth of 32 cm (21 ± 1°C). The water was changed and the instruments were cleaned between each animal test to remove odors. The experiments were videotaped using a numeric tripod-fixed camera for 10 min. The duration of immobility was scored. The time of immobility was measured during 2–6 min of the test, with immobility being defined as a total absence of movement except slight motions to maintain the head above the water. Mice groups were blinded to the observer until the end of analysis.

#### Immunoblotting

The striatum of mice was lysed in CelLytic™ M cell lysis reagent (Sigma-Aldrich, C3228, St Louis, MO, U.S.A) containing protease inhibitors (Sigma-Aldrich, P8340, St Louis, MO, U.S.A) and phosphatase inhibitors (Sigma-Aldrich, P0044, St Louis, MO, U.S.A). The protein concentration was determined using the Bradford method (Bio-Rad, Hercules, CA). Sodium dodecyl sulfate-polyacrylamide gel electrophoresis was performed after loading equal amounts of protein into each lane. The proteins were transferred to polyvinylidene fluoride (Bio-Rad) membranes for immunoblotting. After the membranes were blocked with bovine serum albumin (BSA) (Sigma-Aldrich, A9647, St Louis, MO, U.S.A), they were blotted by adding primary antibodies against DAT (Merck, AB1591P, Darmstadt, Germany) or GAPDH (Genetex, GTX100118, Hsinchu, Taiwan) followed by the appropriate secondary antibodies. The protein bands were detected using an enhanced chemiluminescence kit (PerkinElmer, Waltham, MA) and quantified using 1Dscan Ex gel analysis software (Scanalytics).

## Statistical Analysis

Statistical analysis was performed using the Statistical Package for Social Sciences 23.0 (SPSS Inc., Chicago, IL) and GraphPad Prism version 6.0 (GraphPad Software Inc., La Jolla, CA). In human study, categorical variables were expressed as numbers and percentages, while continuous variables were expressed as the means ± standard deviations (SD) unless otherwise specified. In mice study, results were presented as mean ± standard error of the mean (SEM). Categorical variables were assessed using chi-square tests, while continuous variables were assessed using *t*-tests or one-way ANOVA followed by Tukey’s multiple comparisons. Pearson’s correlations were used to examine the correlations between VPA concentration and the level of striatal DAT availability. The level of significance was set at 0.05 for two-sided tests. The effect size for patient-control differences in striatal DAT was calculated by Hedges’ *g.*

## Results

### The levels of striatal DAT availability were associated with BD and correlated with the VPA concentration in BD patients

We recruited 23 BD patients and 18 control participants. The demographic characteristics are shown in Table 1. Age and gender were not significantly different between the BD patients and the controls. The YMRS and HDRS scores were significantly higher in the BD patients than in the controls (*p* = 0.011 and *p* = 0.001, respectively). In addition, the level of striatal DAT availability was significantly higher in the BD patients than in the controls (right: 1.53 ± 0.18 vs. 1.39 ± 0.26, *p* = 0.045; left: 1.52 ± 0.16 vs. 1.35 ± 0.22, *p* = 0.005; total: 1.52 ± 0.17 vs. 1.37 ± 0.23, *p* = 0.015).

**Table 1.** Demographic characteristics of the BD patients and the control subjects.

	BD patients (*N* = 23)	Controls (*N* = 18)	Comparison			
Characteristics	Mean ± SD	Mean ± SD	*t/χ*^2^	95% CI		*p*
				Lower	Upper	
Age (years)	36.7 ± 12.3	30.8 ± 9.3	1.92	−0.07	13.74	0.052
Gender, female, *n* (%)	15 (65.2)	10 (55.6)	1.77			0.219
YMRS scores	0.8 ± 1.2	0.2 ± 0	2.67	0.19	1.37	0.011*
HDRS scores	2.2 ± 1.2	0.2 ± 0.7	3.59	0.85	3.05	0.001*
VPA concentration (mg/L)	62.3 ± 8.7	–	–	–	–	–
Duration of VPA use (years)	1.8 ± 1.6	–	–	–	–	–
Duration of illness (years)	5.2 ± 4.3	–	–	–	–	–
Previous depressive episodes, *n*	2.9 ± 1.7	–	–	–	–	–
Previous hypomanic episodes, *n*	1.9 ± 1.1	–	–	–	–	–
Proportion of receiving concomitant medications						
Antidepressant, *n* (%)	11 (47.8)	–	–	–	–	–
Benzodiazepine, *n* (%)	15 (65.2)					
Striatal DAT availability						
Right	1.53 ± 0.18	1.39 ± 0.26	2.08	0.01	0.28	0.045*
Left	1.52 ± 0.16	1.35 ± 0.22	2.94	0.05	0.29	0.005*
Total	1.52 ± 0.17	1.37 ± 0.23	2.54	0.03	0.28	0.015*

Abbreviations: BD, bipolar disorder; DAT, dopamine transporter; HAMD, 17-item Hamilton Depression Rating Scale; VPA, valproate; YMRS, Young Mania Rating Scale.

* *p* < 0.05.

**Figure 1.**
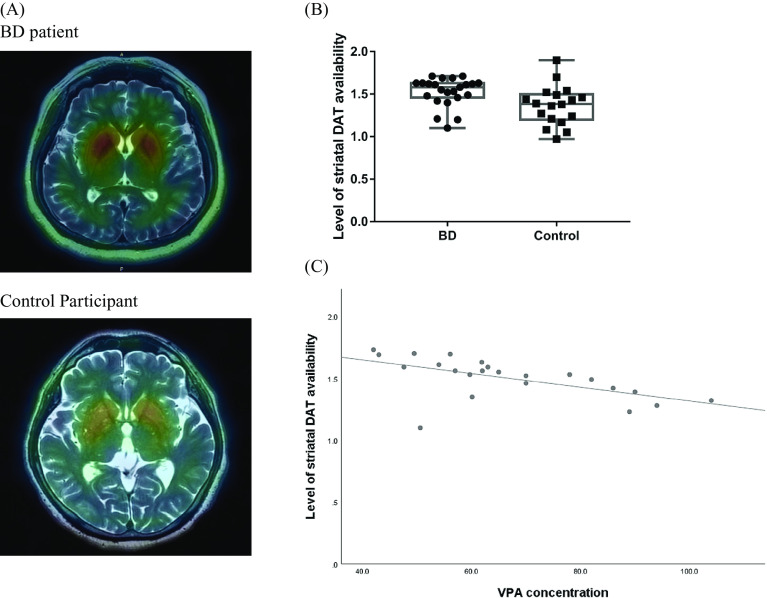
The correlation of the VPA concentration and the level of striatal DAT availability in BD patients. (A) The representative brain image of striatal DAT availability in a BD patient and a control participant. (B) The level of total striatal DAT availability in plots between BD and controls. The effect size was 0.76. (C) The VPA concentration was significantly negatively correlated with the total level of striatal DAT availability in BD patients (*r* = −0.653, *p* = 0.003). Abbreviations: BD, bipolar disorder; VPA, valproate; DAT, dopamine transporter.

Moreover, to investigate whether the level of striatal DAT availability was influenced by the treatment of BD, we further performed correlation analyses (Table 2). The results showed that the VPA concentration was significantly negatively correlated with the level of striatal DAT availability in either the right (*r* = −0.602, *p* = 0.006) or left (*r* = −0.652, *p* = 0.002) sites. Figure 1 showed the correlation of the VPA concentration and the level of striatal DAT availability in BD patients.

**Table 2.** The correlation of the VPA concentration and the level of striatal DAT availability in BD patients.

	VPA concentration (mg/L)	
Striatal DAT availability	*r*	*p*-Value
Right	−0.602	0.006*
Left	−0.652	0.002*
Total	−0.653	0.003*

Abbreviations: BD, bipolar disorder; DAT, dopamine transporter; VPA, valproate.

* *p* < 0.05.

### Alternation of DAT expression after VPA treatment in a chronic SD stress mouse model

**Figure 2.**
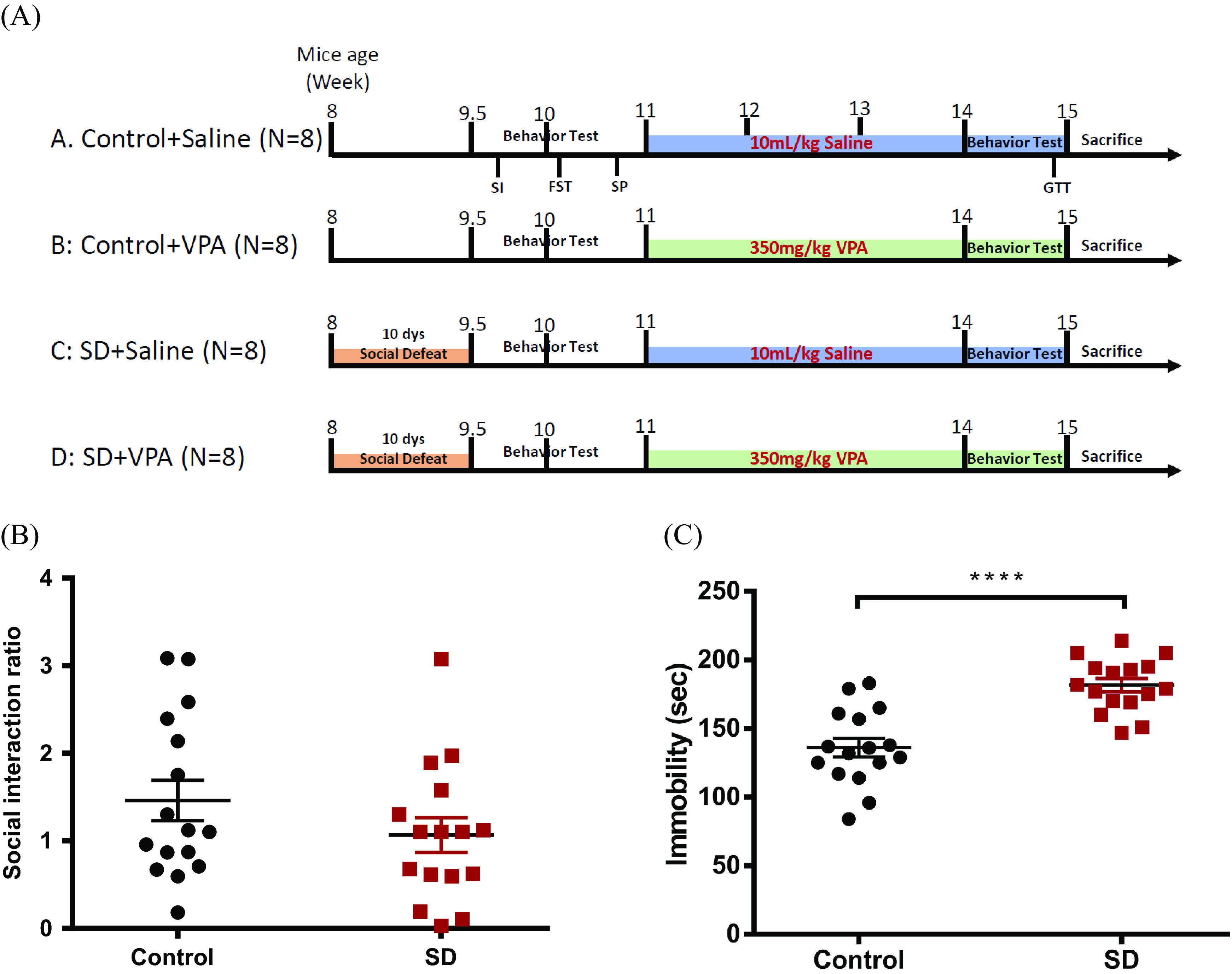
The experimental design of the chronic SD stress mouse model. (A) The scheme showed the experimental design of the chronic SD stress mouse model. (B) After SD, the social interaction ratio was decreased in SD mice group (*N* = 16) than that in control (*N* = 16). (C) The immobility of FST was increased in SD mice group than that in control (*p* < 0.0001). Data were presented as mean ± SEM (*N* = 16 per group). ****p < 0.0001. Abbreviations: SD, social defeat; VPA, valproate.

To investigate the observational association between VPA and striatal DAT levels, we used an SD mouse model that showed BD depression-like behavior. The 8-week-old mice were social defected for 10 days and then analyzed for their indices at baseline and after VPA treatment (350 mg/kg) or normal saline (10 mL/kg) for 3 weeks (Figure 2A). After SD, the ratio of the social interaction score was decreased, and the immobility of the FST was increased, both of which represented depression-like behavior, in the SD mouse group compared with the control group (Figure 2B,C). Body weight and food intake were not significantly different between the control and SD groups with or without VPA (Supplementary Figure 1).

**Figure 3.**
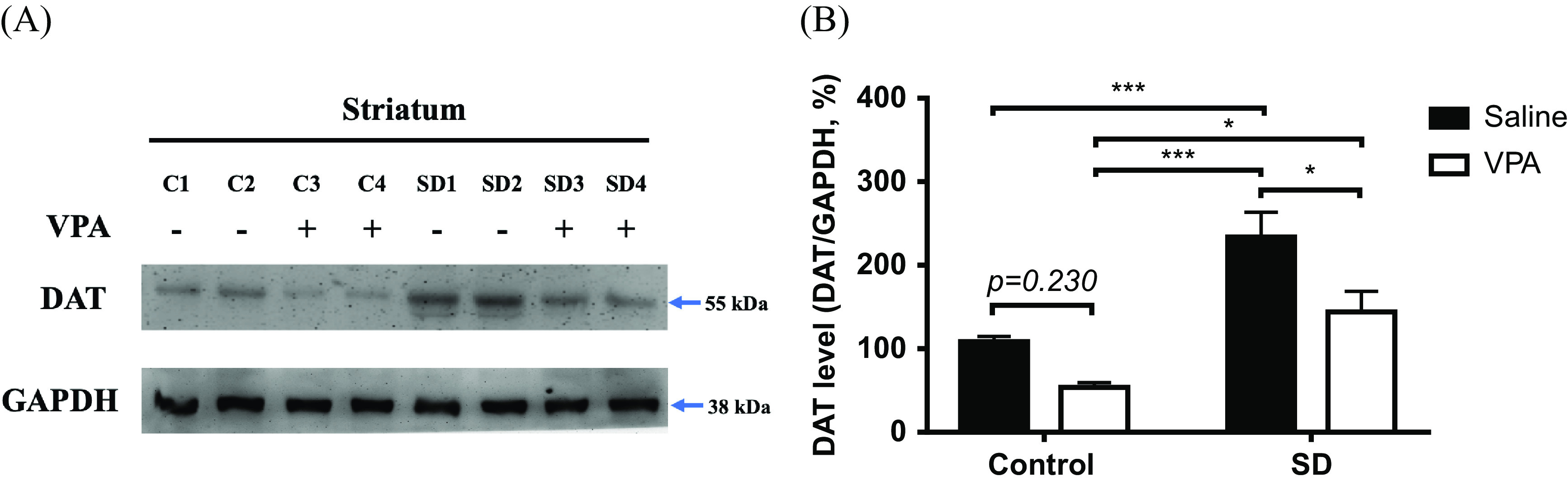
The DAT expression at striatum in chronic SD stress mouse model treated with VPA. (A) Representative western blot reveals the expression levels of DAT at striatum in mice. (B) Quantification of western blot data. Each group contained eight independent samples. The result indicated that the DAT level at striatum significantly increased in SD group compared to control group (indicated all of data sub-grouped by control (*N* = 16) or SD mice group (*N* = 16), *p* < 0.001). Considering the effect of 3-week VPA treatment, the DAT expression significantly decreased in SD + VPA group compared with the SD + saline, while there was not different between control + VPA and control + saline (*p* = 0.230). Moreover, although the reduced level of DAT expression in SD + VPA group was significantly higher than that in control + VPA (*p* < 0.05), the levels were not significantly different between the SD + VPA and control + saline groups. Data were expressed as mean ± SEM (*N* = 8 per group). *p < 0.05, ***p < 0.001 Abbreviations: SD, social defeat; VPA, valproate; DAT, dopamine transporter.

In addition, the expression of DAT in the striatum (Figure 3A) was significantly increased in the SD group compared to the control group (SD effect, *p* < 0.001). Considering the effect of 3 weeks of VPA treatment, DAT expression was significantly decreased in the SD + VPA group compared with the SD + saline group, while there was no difference between the control + VPA and control + saline groups (*p* = 0.230) (Figure 3B). Moreover, although the reduced level of DAT expression in the SD + VPA group was significantly higher than that in the control + VPA group (*p* < 0.05), the levels were not significantly different between the SD + VPA and control + saline groups (Figure 3B). The results suggested that VPA treatment might rescue DAT expression to baseline in mice under chronic stressful conditions.

## Discussion

To our knowledge, this was the first clinical study to clarify the association between striatal DAT and VPA levels in euthymic BD. In the current study, we found that although there was a negative correlation between the VPA concentration and the level of striatal DAT availability, an increased level of striatal DAT availability still existed in BD patients treated with VPA who were in a euthymic stage compared with controls. In addition, we also demonstrated that SD mice had increased expression of DAT in the striatum and that SD mice treated with VPA significantly decreased DAT expression to baseline levels similar to those in control mice treated with saline. Thus, the results of this study suggested that DAT availability plays an important role in BD pathophysiology, and the homeostasis of DAT availability might be a new therapeutic strategy for BD patients.

Recently, the dopamine hypothesis has been proposed to be an important theory in the neurobiological pathophysiology of BD [5, 19]. The dysregulation of homeostasis in response to hyperdopaminergia in the manic phase of the illness results in an excessive reduction in dopaminergic function, leading to a hypodopaminergic stage and depression, and vice versa [5]. In animal studies, the inhibition of DAT activity by gene knockout or DAT blockers has been shown to induce manic-like behavior, such as hyperlocomotion and increased exploration [20,21], while using quinpirole (a dopamine agonist) was also shown to induce manic-like behavior [22]. In contrast, lesions in dopaminergic areas induce depressive-like behavior in a rodent model [23,24]. However, in human imaging studies of mania, the availability of the striatal D2/3 receptor density was not associated with nonpsychotic mania in BD patients using [^11^C]N-methylspiperone [25,26], and dopamine synthesis was not correlated with mania severity [27,28]. Interestingly, D2/3 receptor density was found to be correlated with psychotic mania symptoms in BD [26], which was similar to schizophrenia. In depressive BD patients, the DAT availability in the caudate nucleus was lower than that in controls [29], while the findings remain controversial [5]. Although the evidence indicates that homeostasis of the dopamine system is involved in BD, there have been limited studies on the euthymic stage [6,27,30]. There was no difference in striatal D2 receptor binding and no alteration in the dopamine release paradigm when compared with controls [30]. However, this is not consistent with the finding of DAT availability. The upregulation of DAT availability in the striatum in drug-naive euthymic bipolar patients [6] was similar to our results of the current study in euthymic BD patients treated with VPA. In contrast, Anand et al. observed a downregulation of DAT at the dorsal caudate in unmedicated euthymic BD patients, and there was no difference in the striatum [27]. The difference might be affected by several confounding factors, such as medication status, clinical features, duration of illness, numbers of episodes, types of BD, and substance abuse. Taken together, although implicit in the dopamine hypothesis suggests that normalization of dopaminergic function leads to remission and euthymia, further research is still needed to determine the key components of the dopaminergic circuit in BD.

The therapeutic action of medications for the treatment of BD might involve modulation of the dopamine system [11]. Mood stabilizers, including lithium and VPA, exhibit therapeutic effects on the dopaminergic system. Lithium could modulate downstream dopamine receptors in postsynaptic dopaminergic signal transduction by acting through the AKT/GSK3 signaling cascade [31]. In addition, VPA treatment reduced presynaptic dopamine synthesis capacity [28], but D2/3 density remained unchanged [10]. This suggests that VPA might block the capacity of the D2/3 receptor to respond to reduced dopamine synthesis or that the capacity of D2 receptors to respond is intrinsically impaired in BD. Furthermore, in our current study, we found that VPA treatment could significantly decrease the elevated DAT under BD conditions in both human and SD mouse model studies. Similar results were found that VPA exerted beneficial effects on mood by normalizing the lengthened circadian rhythm period in participants with elevated dopamine resulting from reduced DAT expression using a DAT-deficient Drosophila or DAT-KD mouse model [32]. The possible mechanism of VPA-affected DAT might involve histone acetylation and d promoter binding of Nurr1 [33], although these findings have not been consistent. In addition to mood stabilizers used to treat BD, certain antipsychotics, including olanzapine, quetiapine, ziprasidone, and long-acting injections aripiprazole and risperidone, have been approved for the maintenance treatment of BD either as monotherapy or as adjunctive therapy [34]. The blockade of dopamine D2/3 receptors is their common mechanism of action. Taken together, a growing body of evidence indicates that regulation of the dopamine system partially explains the therapeutic efficacy of BD. It not only suggests dopaminergic drugs have a place in BD but also indicates the target of the dopamine system noted as a therapeutic strategy. Whether striatal DAT availability might be a marker of BD treatment response is of interest. Therefore, longitudinal molecular imaging studies investigating dopaminergic function, particularly DAT and D2/3 receptor homeostasis, in patients across different illness phases are needed. Moreover, it is also interesting to investigate the effects of antidopaminergic and mood stabilizers on dopaminergic pathways and the relationship between dopaminergic blockade and treatment response.

There were some limitations of the present study. First, although there was a relatively small sample size of BD patients and community controls from a single site, the power was 0.81. Second, since we focused on BD treated with VPA at the euthymic stage in the current study, the results cannot be extrapolated to other BD patients in different mood stages, such as depression or mania. A longitudinal study that investigated changes across phases of BD can fully elucidate phase-related dopamine dysfunction, but it is difficult due to clinical practice. Third, we did not collect information on factors such as diet and exercise that may influence DAT availability in BD patients and community controls, although we have used the SD mouse model to validate the results. Fourth, we focused on BD patients at euthymic stage, while used chronic SD stress mouse model, mimicking BD depression, to test whether striatal DAT expression changes after VPA treatment. The mood stage is different, but there is no mouse model to mimic BD at euthymic stage. Fifth, since our study focused on the association among DAT availability, BD, and VPA treatment, whether dopaminergic dysregulation is linked to the involvement of other neurotransmitter systems is not clear. Therefore, further larger sample sizes and mechanisms should be employed to confirm our results.

In conclusion, this was the first clinical study to clarify the negative correlation between VPA concentration and the level of striatal DAT availability in euthymic BD patients treated with VPA, while an increased level of striatal DAT availability existed in BD patients compared to controls. Our novel results suggest that the DAT system, particularly DAT availability, plays an important role in the pathophysiology of BD and is involved in VPA-mediated physiological alterations. Therefore, DAT availability could be a mediator of a shared mechanism of BD and the therapeutic action of medications. Furthermore, homeostasis might represent a new therapeutic strategy for BD patients.

## Data Availability

The data that support the findings of this study are available from National Cheng Kung University. Restrictions apply to the availability of these data, which were used under license for this study. Data are available for Hui Hua Chang with the permission of National Cheng Kung University.
